# Supplementary material for: Effectiveness of Prophylactic Human Cytomegalovirus Hyperimmunoglobulin in Preventing Cytomegalovirus Infection following Transplantation: A Systematic Review and Meta-Analysis
Source: Life (Basel). 2022 Mar 2;12(3):361. doi: 10.3390/life12030361 (PMC8955988; doi:10.3390/life12030361)
Supplement: Supplementary file 1 [file life-12-00361-s001.zip › life-1590276-supplementary.pdf]

Table S1. Study Characteristics.

Study	Transplant type	Study design	Patient serological status	Treatment (n)	Control (n)	Timing of treatment	Estimated cumulative dose per 70 kg patient	Method of assessing CMV infection	Immuno-suppression	Rejection rate	Time to infection	AEs
Varga et al. (2005) [54]	Renal	Retrospective<br>Arms:<br>• Cytotect<br>• Cytotect + ganciclovir<br>• Ganciclovir<br>• No prophylaxis	All D+/R−	Cytotect (53)<br>Cytotect + ganciclovir (30)	No prophylaxis (22)<br>Ganciclovir (42)	Cytotect: 1 mL/kg IV administered on days 0, 14, 35, 56, 77, and 98 post transplantation<br>Cytotect + ganciclovir: 2 doses Cytotect (1 mL/kg IV) on days 0 and 14 post transplantation<br>Ganciclovir: 1.5 g/day during the first 3 months post transplantation<br>Ganciclovir: 3 g/day for 3 months from day 14 post transplantation	420 mL Cytotect only or 140 mL Cytotect + ganciclovir	Detection of CMV pp65 antigen (antigenemia test) in circulating peripheral blood leukocytes	Cytotect: CsA + steroids<br>Cytotect + ganciclovir: CsA + MMF + steroids<br>No prophylaxis: CsA + steroids<br>Ganciclovir: Tacrolimus + MMF + steroids	In first year<br>Cytotect: 18.9%<br>Cytotect + ganciclovir: 13.3%<br>No prophylaxis: 22.7%<br>Ganciclovir: 14.3%	[% within 4 months]<br>Cytotect: 59%<br>Cytotect + ganciclovir: 23%<br>No prophylaxis: 100%<br>Ganciclovir: 14.3%	NR
Wirnsberger et al. (1999) [56]	Renal	Prospective, controlled, and randomized<br>Arms:<br>• Cytotect<br>• No prophylaxis	20 D+/R−; 11 D+/R+; 32 D−/R+; 11 D−/R−	Cytotect (38)	No prophylaxis (36)	2 mL/kg IV before transplantation and on days 1, 2, 4, 18, 32, 46, 60, 74, and 88 post transplantation	1400 mL	Positive CMV pp65 assay and/or an IgG or IgM seroconversion	Induction: steroids<br>Maintenance: CsA	Cytotect: 44.7%<br>Control: 44.4%	NR	NR
Ginevri et al. (1998) [31]	Renal (pediatric)	Retrospective, controlled<br>Arms:<br>• R−: CMV prophylaxis with acyclovir + Cytotect<br>• R+: CMV prophylaxis with acyclovir<br>In the presence of CMV infection, ganciclovir was administered	33 D+/R−; 28 D+/R+; 12 D−/R+; 6 D−/R−	Cytotect + acyclovir (39)	Acyclovir (40)	Cytotect: 150 mg/kg on the first day post transplantation, 100 mg/kg on days 15 and 30, and 50 mg/kg on days 45, 60, and 120. Starting in February 1995, 150 mg/kg was given on the first day post transplantation and twice monthly for 2 months; 100 mg/kg was given in the third and fourth months<br>Acyclovir: 40 mg/kg per day until January 1995 and then 80 mg/kg per day	35 g; 66.5 g starting in February 1995	Detection of CMV pp65 antigen in circulating<br>peripheral blood leukocytes	CsA, steroids (+ AZA in 4 patients receiving from living donor)	Acute rejection rate:<br>CMV infected: 33.3%<br>Not CMV infected: 51%	Median time to onset of CMV infection:<br>48 ± 4.1 days (range 14 to 105 days)	No AEs requiring discontinuation
Pakkala et al. (1992) [42]	Renal	Prospective, controlled, and randomized<br>Arms:<br>• Cytotect<br>• CMVIG (Finnish)<br>• No prophylaxis	All D+/R−	Cytotect (15)<br>CMVIG (15)	No prophylaxis (15)[a]	Cytotect or Cytogam 1.5 mL/kg on day 1, 1.0 mL/kg on days 14 and 28, and 0.5 mL/kg on days 42, 56, 84, and 112 post transplantation	385 mL (19, 250 U)	CMV rapid shell vial cultures from urine and blood, performed monthly, and immunodetection of CMV-specific immediate early antigens in symptomatic patients	AZA, CsA + steroids	Cytotect: 33.3%<br>CMVIG: 40%<br>Control: 47%<br>Rejection was uncorrelated to CMV in all groups	NR	NR

Metselaar et al. (1989) [41]	Renal	Prospective, controlled, and randomized<br>Arms:<br>• Cytotect<br>• Placebo	8 D−/R−; 9 D+/R−; 22 D+ or D−/R+	Cytotect (19)	Placebo (20)	100 mg/kg on the day of ATG treatment and on days 7, 14, 21, 35, 56, and 77	42 g	CMV from peripheral blood leukocytes, urine, or throat wash was detected by immunofluorescence or by cytopathic changes when cultured with embryonic lung fibroblasts	CsA + steroids	Rejection was entry criteria for study	NR	NR
Fassbinder et al. (1985) [29]	Renal	Prospective, controlled, and randomized<br>Arms:<br>• Cytotect<br>• IgG (Intraglobin)	40 R+; 43 R−; donor status not reported	Cytotect (42)	IgG (34)	10 g Cytotect or IgG immediately before transplantation and on days 18, 38, 58, and 78 post transplantation	50 g	Presence of CMV antibodies (detected by ELISA) in a previously seronegative patient or a 4-fold increase in CMV titer in a previously seropositive patient	AZA + steroids<br>Rejection treatment: 2–4 bolus dosages (1 g each) of prednisolone<br>For steroid-resistant patients: plasmapheresis or ATG (per histology)	NR	89% within 3 months	No AEs
Greger et al. (1986) [32]	Renal	Prospective, controlled, and randomized<br>Arms:<br>• CMVIG (product unspecified) + CsA<br>• CMVIG (product unspecified) + AZA + ATG<br>• CsA (no CMVIG)<br>• AZA + ATG (no CMVIG)	NR	CMVIG (24)	No CMVIG (24)	0.1 g/kg IV twice weekly immediately before transplantation, on day 1 post transplantation, and every 3 weeks for the first 6 months post transplantation	63 g	Complement fixation assays and CMV-IGM-ELISA tests in sera collected prior to each treatment. A 4-fold increase in either test was diagnosed as an infection	CsA + steroids or AZA + ATG + steroids	NR	NR	No AEs
Snydman et al. (1987) [50]	Renal	Prospective, controlled, and randomized<br>Arms:<br>• CMVIG (Cytogam)<br>• No CMVIG	All D+/R−	Cytogam (24)	no CMVIG (35)	150 mg/kg within 72 hours of transplantation, 100 mg/kg 2 and 4 weeks post transplantation, and 50 mg/kg 6, 8, 12, and 16 weeks post transplantation	38.5 g	CMV viremia (viral isolation from peripheral blood leukocytes), CMV isolation from urine and throat wash (per culture on human skin fibroblasts and embryonic kidney cells), CMV seroconversion per antibody measurement by indirect hemagglutination in sera, confirmed in CMVIG-treated patients by CMV-specific IgM immunoassay	CsA and/or AZA + steroids ± ALG or CsA + AZA + steroids	Cytogam: 15%<br>Control: 54%	Cytogam: 49 days (range 33–187 days)<br>Control: 42 days (range 15–365 days)	Flushing (n=3)<br>Anxiety, nausea, metallic taste, headache, shortness of breath (all n=2)<br>Palpitations, backache, muscle cramp (all n=1)

Grundmann et al. (1987) [33]	Renal	Prospective, controlled, and randomized<br>Arms:<br>• CMVIG (Polyglobin)<br>• No CMVIG	NR	Polyglobin (50)	No CMVIG (50)	2 mL/kg immediately before transplantation and on days 1, 21, 42, 63, 84, and 105 post transplantation	840 mL	Complement fixation reaction and CMV IgM titers were determined by ELISA. Infection was diagnosed based on a 4-fold increase in complement fixation reaction or an IgM titer >1:10 on ELISA	Induction: ALG + AZA + steroids<br>Maintenance: CsA + steroids	NR	NR	NR
Tenschert et al. (1993) [53]	Renal	Retrospective data<br>Arms:<br>• Cytotect<br>• No prophylaxis	All D+/R−	Cytotect (18)	No prophylaxis (18)	2–3 mL/kg immediately before transplantation and on day 1, then twice a week for up to 10 weeks[b]	Up to 4400 mL	pp65/p72 shell vial assay	CsA + AZA + steroids<br>Rejection: steroids<br>Steroid-resistant: ATG	Vascular rejection<br>Cytotect: 27.8%<br>Control: 22.2%	Cytotect: 42 days ± 3<br>Control: 31 days ± 12	NR
Werner et al. (1993) [55]	Renal	Retrospective data of D+/R− patients	All D+/R−	Cytogam (174)	No control	150 mg/kg within 72 hours; 100 mg/kg at 2, 4, 6, and 8 weeks; 50 mg/kg at 12 and 16 weeks	45.5 g	Viral isolation, IgM antibody to CMV, or PCR	CsA + AZA + steroids (53%)<br>OKT3 or ALS + AZA + steroids ± CsA (40%)	25%		AEs included flushing, tachycardia, back pain, chest tightness, myalgia, arthralgia, increased temperature, and increased BP; 7 patients in total had AEs
Flechner et al. (1998) [30]	Renal	Prospective data (collected for comparison acyclovir vs GCV)<br>Arms:<br>• Cytogam + acyclovir or ganciclovir<br>• Acyclovir or ganciclovir	27 D+/R−; 29 D+/R+; 23 D−/R+; 22 D−/R−	D+/R− (high risk):<br>Cytogam + acyclovir or ganciclovir (27)	D+ and/or R+<br>Acyclovir or ganciclovir (52)	Every other week for 16 weeks	NR	Isolation of CMV from fluids (apart from urine) or tissues	Induction: OKT3<br>Maintenance: CsA + steroids +AZA or MMF<br>Rejection: steroids<br>Steroid-resistant: OKT3 or ATG	29.6% in Cytogam + acyclovir or ganciclovir group	NR	NR
Snydman et al. (1991) [51]	Renal	Prospective Open-label trial	All D+/R−	Cytogam (36)		150 mg/kg within 72 hours and at 2, 4, 6, and 8 weeks; 100 mg/kg at 12 and 16 weeks	45.5 g	Not defined	CsA or AZA + steroids ± ALS or CsA + AZA + steroids	61%	NR	5.2% had AEs possibly due to treatment, including chest tightness, muscle cramps, back pain, flushing, and chills

Stratta et al. (1994) [52]	Pancreas and kidney	Prospective<br>Arms:<br>• Cytogam + ganciclovir + acyclovir<br>• Ganciclovir + acyclovir	6 D+/R−; 44 D+/R+; 27 D−/R+; 5 D−/R−	Cytogam (9)	Ganciclovir + acyclovir (34)	150 mg/kg at 48–72 hours and 100 mg/kg at 2, 4, 6, and 8 weeks	38.5 g	Anti-CMV antibody titers were measured by indirect immunofluorescence and considered positive in the presence of CMV-IgM or a 4-fold increase in CMV-IgG titers. Body fluid samples	Induction: OTK3 + CsA + AZA + steroids	Cytogam + ganciclovir + acyclovir: 55.5%<br>Ganciclovir + acyclovir: 65.5%	Cytogam + ganciclovir + acyclovir: 110 days ± 45<br>Ganciclovir + acyclovir: 55 days ± 12	NR
Boland et al. (1993) [26]	Heart and renal	Prospective, controlled<br>Arms:<br>• Cytotect<br>• No prophylaxis	All D+/R−	Cytotect (14)	No prophylaxis (14)	1 mL/kg IV 1, 2, 3, 5, and 7 weeks post transplantation	35 g	Antigenemia and/or positive cultures with anti-CMV IgG/IgM antibodies detected in serum using ELISA	CsA + steroids or AZA (steroids for kidney transplant, triple treatment for heart transplant)<br>Rejection treatment: high-dose steroids; in severe/persistent: OKT3 or ATG	Cytotect: 35.7% within 6 weeks<br>Control: 42.9% within 6 weeks	NR	NR
Kocher et al. (2003) [36]	Heart	Retrospective, uncontrolled<br>Single arm: Cytotect	93 D+/R−; 134 D+/R+; 76 D−/R+; 74 D−/R−	Cytotect (377)		1.5 mg/kg IV before transplantation and 1, 7, 14, 21, and 28 days post transplantation	0.5 g	Evidence of CMV immediate early antigen in cultures of blood, urine, or throat wash or in biopsy samples	Induction: ATG for 7 days post transplantation<br>Maintenance: CsA + AZA + steroids	NR	22.8% within 1 week	No CMVIG AEs;<br>18.8% discontinued ATG after day 4 due to thrombocytopeni a or leukopenia
Metselaar et al. (1990) [40]	Heart	Retrospective, controlled<br>Arms:<br>• Cytotect<br>• No prophylaxis	18 D+/R−; 16 D−/R−; 38 D+ or D−/R+	Cytotect (32)	No prophylaxis (40)	150 mg/kg during transplantation, then 100 mg/kg on days 2, 7, 14, 35, 56, and 77 post transplantation	52.5 g	Serum screened for anti-CMV IgG using ELISA. Blood, urine, and throat wash samples were collected for viral isolation	CsA, steroids<br>Rejection treatment: bolus steroids<br>Steroid-resistant: ATG	NR	NR	6.3% (n=2) discontinued Cytotect due to rash
Eisenmann et al. (1990) [28]	Heart	Prospective, uncontrolled<br>Single arm: Cytotect	13 D+/R+; 5 D+/R−; 4 D−/R+; 1 D−/R−	Cytotect (23)		1 mL/kg on days 1, 8, 15, 22 and week 6 post transplantation	350 mL	CMV antibody titers analyzed weekly using ELISA	Triple treatment: CsA + AZA + steroids	NR	NR	NR
Balk et al. (1993) [25]	Heart	Prospective, controlled<br>Arms:<br>• R−: Cytotect<br>• R+: No CMVIG prophylaxis	34 R−/D−; 29 R−/D+; 2 R−/D unknown; 81 R+/D+ or D−	Cytotect (65)	No CMVIG prophylaxis (81)	150 mg/kg during the operation and 100 mg/kg 2, 7, 14, 28, 56, and 72 days post transplantation	59.5 g	Appearance of IgM, isolation of CMV from urine, throat wash, or blood, or any demonstration of the antigen	CsA + prednisone (+ AZA in 15 patients)<br>Early rejection prophylaxis: CsA + steroids (n=55); ALG (n=34); OTK3 (n=57)<br>Acute rejection: pulsed steroids or ATG<br>Other rejection: OTK3	Mean 1.4 rejection episodes in first year, 1.5 after first year<br>No difference between R− and R+	R−: 8.9 weeks<br>R+: 9.7 weeks	Transient rash in 4.6% (n=3)

Havel et al. (1989) [34]	Heart	Prospective, uncontrolled<br>Single arm:<br>• Cytotect	46 R+; 4 R−; donor status not reported	Cytotect (50)		1 mL/kg administered on the day of the transplant and weekly until day 30 post transplantation	38.5 g	CMV-specific IgG antibodies were assayed by complement-binding reaction, and IgM antibodies were assayed by ELISA	Pre-op: CsA + AZA<br>Intra-op and early post-op: steroids<br>Days 1–10: ATG or OKT3 + CsA, AZA, steroids<br>After day 10: CsA, AZA, steroids	NR	NR	No AEs
Yamani et al. (2005) [57]	Heart	Prospective, controlled, and randomized (except in those receiving no intervention)<br>Arms:<br>• Cytogam<br>• Placebo/no intervention	10 D+/R−; 18 D+/R+; 19 D−/R+; 9 D−/R−	Cytogam (13)	Placebo (10)<br>No intervention (33)	150 mg/kg IV over 4 hours. Another dose was administered 4 weeks later if the IgG level was <500 mg/dl (mean 1.4 doses/patient)	14.7 g	Positive CMV DNA test by quantitative PCR with clinical manifestations suggestive of a syndrome	MMF + CsA or AZA + CsA or MMF + tacrolimus or rapamycin + CsA<br>All + steroids	Average acute cellular rejection ≥grade 2<br>Cytogam: 0.4<br>Control: 1.4	NR	No AEs
Lopez-Garcia et al. (2015) [38]	Lung	Retrospective, controlled<br>High-risk: D+/R−<br>Low-risk: D+ or D−/R+<br>Arms:<br>• IV ganciclovir 1 week followed by oral valganciclovir<br>• Cytotect (switched to Cytotect monotherapy in patients with persistent leukopenia)	26 D+/R−; 133 D+ or D−/R+	Cytotect + ganciclovir + valganciclovir (23)	Ganciclovir + valganciclovir (133)	D+/R– and R+ patients:<br>ganciclovir (5 mg/kg) for 1 week, then valganciclovir for 3 months (R+) or 6 months (R–)<br>D+/R– patients: Cytotect (2 mg/kg) on days 1, 4, 8, 15, and 30 post transplantation, then monthly for an additional year	2.2 g	CMV pp65 antigenemia for part of the study, after which quantitative PCR of plasma was used	Induction: basiliximab<br>First month: tacrolimus + MMF + steroids	Acute rejection rate<br>D+/R−: 38%<br>R+: 30%	NR	NR
Ruttmann et al. (2006) [46]	Lung	Retrospective, controlled<br>Arms:<br>• Cytotect + ganciclovir<br>• Ganciclovir	23 D+/R−; 45 D+/R+	Cytotect + ganciclovir (38)	Ganciclovir (30)	Cytotect: 1 mL/kg 1, 3, 5, 7, 14, 21, 28 days post transplantation<br>Ganciclovir: 10 mg/kg IV daily at days 1–14 followed by oral ganciclovir 3 g daily or valganciclovir 900 mg twice daily until 100 days post transplantation	49 g	pp65 CMV antigenemia test	Intra-op: steroids<br>Maintenance: CsA + AZA + MMF + steroids<br>19 patients in control group received ATG, 49 patients received daclizumab	Acute rejection in first year:<br>Cytotect: 47.5%<br>Control: 58.3%	In first year:<br>Cytotect: 28.5%<br>Control: 48.9%	NR

Weill et al. (2003) [24]	Lung	Retrospective<br>Arms:<br>• CMVIG (unknown) + ganciclovir<br>• Ganciclovir	16 D+/R−; 45 D+/R+; 25 D−/R+	CMVIG + ganciclovir (38)	Ganciclovir (48)	CMVIG + ganciclovir<br>R+: 3 doses of CMVIG (1 dose every 2 weeks) + 6 weeks ganciclovir<br>R−: 12 weeks ganciclovir<br>Ganciclovir<br>R+: 6 weeks ganciclovir<br>R−: 12 weeks ganciclovir<br>CMVIG dosed at 150 mg/kg within 72 hours post transplantation, then every 2 weeks for 4 doses, then 100 mg/kg every 4 weeks for 2 additional doses	66.5 g	Positive CMV antigenemia in blood specimens or a positive CMV buffy coat	Induction: Both groups received steroids<br>CMVIG + ganciclovir group also received daclizumab<br>Maintenance: CsA + AZA + steroids<br>Acute cellular rejection: steroids	Acute rejection in first 6 months<br>CMVIG + ganciclovir: 66%<br>Ganciclovir: 79%	CMVIG + ganciclovir: 148 days ± 14.9<br>Ganciclovir: 92 days ± 26.4	NR
Kruger et al. (2003) [37]	Lung	Prospective, randomized data<br>Arms:<br>• Cytogam<br>• No prophylaxis	28 D+/R+; 16 D−/R+	Cytogam (22)	No prophylaxis (22)	150 mg/kg within 72 hours of transplantation; 150 mg/kg at 2, 4, 6, and 8 weeks after transplantation; 100 mg/kg at 12 weeks after transplantation	59.5 g	Positive shell vial assay or the isolation of CMV in conventional cell culture. Follow-up weekly for 12 weeks and at 6 and 12 months	Induction: CsA + AZA + ATG or basiliximab + steroids<br>Maintenance: CsA + AZA + steroids	Acute rejection ≥grade A2 or B1 (episodes/patient)<br>Cytogam: 1.00 ± 0.98<br>Control: 1.09 ± 1.5	Cytogam: 44 days ± 18<br>Control: 41 days ± 15	1 patient experienced mental deterioration that was fully resolved; association with Cytogam uncertain
Kathawalla et al. (1996) [35]	Lung	Retrospective data<br>Arms:<br>• With CMVIG<br>• Without CMVIG	All D+/R−	CMVIG (3)	No CMVIG (6)	Not defined	NR	Not defined	Not defined	CMVIG: 33.3%<br>No CMVIG: 66.6%	CMVIG: 79 days<br>No CMVIG: 69 days	NR
Ranganathan et al. (2009) [44]	Lung	Retrospective data<br>Arms:<br>• CMVIG + ganciclovir<br>• Ganciclovir only	133 D+/R; 77 D+/R+; 53 D−/R+; 57 D−/R−	CMVIG + ganciclovir (49)	Ganciclovir only (220)	Dosing regimens for CMVIG varied widely. Patients received a mean of 5 doses of CMVIG (range 1–12). Dosing interval was most commonly every 2 weeks (range 1 day–1 month). Median duration of therapy after transplantation was 84 days (range 1–192 days), and median dose was 150 mg/kg (mean 133 mg/kg)		Conventional viral culture, shell vial viral culture, pp65 antigenemia testing, or CMV PCR in whole blood or peripheral blood mononuclear cells	CMVIG + ganciclovir: MMF in 31% of patients	NR	CMVIG + ganciclovir: 85 days<br>Ganciclovir only: 87 days	No complications reported
Snydman et al. (2001) [47]	Liver	Prospective with historic control (patients participating in previous trials)<br>Arms:<br>• CMVIG (Cytogam) + ganciclovir<br>• Placebo or CMVIG	All D+/R−	Cytogam + ganciclovir (39)	Placebo or CMVIG (47)	Cytogam: 150 mg/kg within 72 hours of transplantation and 2, 4, 6, and 8 weeks post transplantation; 100 mg/kg 12 and 16 weeks post transplantation<br>Ganciclovir: 5 mg/kg intravenously twice a day for 2 weeks after transplantation	66.5 g	Complement fixation, latex agglutination, indirect hemagglutination, and ELISA<br>Seroconversion confirmed by CMV-specific IgM antibody immunoassay	CsA or tacrolimus + AZA + steroids<br>Acute rejection: bolus IV steroids<br>Steroid-resistant: OKT3	Cytogam + ganciclovir: 15%<br>Placebo or CMVIG: 47%	Cytogam + ganciclovir: 55 days (range 52–66 days)<br>Placebo or CMVIG: 34 (range (27–39 days)	Back pain (n=4)<br>Chills (n=4)<br>Flushing (n=2)<br>Anxiety (n=2)<br>Decrease in BP (n=2)<br>Long bone pain (n=1)

Rabkin et al. (2001) [43]	Liver	Retrospective with historical control<br>Arms:<br>• CMVIG + acyclovir<br>• Acyclovir only	All D+/R−	CMVIG + acyclovir (7)	Ganciclovir (11)<br>Acyclovir only (15)	150 mg/kg within the first 72 hours after transplantation followed by 100 mg/kg every 2 weeks for 4 doses and 50 mg/kg at weeks 12 and 16 in addition to the acyclovir protocol	45.5 g	Post-transplant CMV infection was defined as the clinical syndrome of leukopenia, fever, and myalgia, requiring treatment in the presence of CMV positive shell vial culture from blood, urine, or sputum	Induction: anti-lymphocyte agents (not specified)<br>Maintenance: calcineurin inhibitors (not specified) + purine antagonists (not specified) + steroids<br>Rejection: anti-lymphocyte agents<br>Steroid-resistant: OKT3	NR	CMVIG + acyclovir: 48 days<br>Acyclovir: 62 days<br>Ganciclovir: 399 days	NR
Snydman et al. (1993) [49]	Liver	Prospective, randomized, placebo-controlled<br>Arms:<br>• Cytogam<br>• Placebo	38 D+/R−;<br>25 D+/R+;<br>44 D−/R−;<br>34 D−/R+	Cytogam (69)	Placebo (72)	150 mg/kg within 72 hours and at 2, 4, 6, and 8 weeks, then 100 mg/kg at 12 and 16 weeks	66.5 g	CMV antibody was measured by complement fixation, latex agglutination, indirect hemagglutination, and ELISA. Seroconversion confirmed by testing for CMV-specific IgM antibody by enzyme immunoassay	CsA + AZA + steroids (+ OKT3 in 35% patients)<br>Rejection: bolus steroids<br>Refractory or recurrent: OKT3	Rejection episodes<br>Cytogam:<br>0%–26%<br>1%–46%<br>>1%–27%<br>Placebo:<br>0%–25%<br>1%–42%<br>>1%–33%	Cytogam: 45.3 days ± 71.3<br>Placebo: 41.9 days ± 38.0	AEs in 6.7% Cytogam vs 3.8% placebo;<br>Most common: Pain, fever, flushing, increased BP
Snydman et al. (1994) [48]	Liver	Prospective<br>Open-label trial<br>Single arm: Cytogam	6 D+/R−;<br>44 D+/R+;<br>27 D−/R+;<br>5 D−/R−	Cytogam (21)		150 mg/kg within 72 hours and at 2, 4, 6, and 8 weeks; 100 mg/kg at 12 and 16 weeks	38.5 g	CMV antibody was measured by complement fixation, latex agglutination, or ELISA	CsA + AZA + steroids	Rejection episodes<br>0%–24%<br>1%–43%<br>>1%–33%	28 days (range 9–40 days)	NR
Kornberg et al. (2020) [58]	Liver	Retrospective, controlled<br>Arms:<br>• Cytotect (D+/R−, D+/R+, D−/R+)<br>• No prophylaxis (D−/R−)	14 D+/R−;<br>4 D+/R+;<br>7 D−/R+;<br>18 D−/R−	Cytotect (25)	No prophylaxis (18)	Cytotect: 1 mL/kg/day; infusion rate 0.8 mL/kg/h; started at day 1 post-transplantation and continued for a minimum of 1 week.	Min. 490 ml	Positive CMV DNAemia in serum at any level, measured by quantitative PCR	Tacrolimus + steroids	Biopsy-proven allograft rejection<br>Cytotect: 0%<br>Control: 4%	[% at 3 months]<br>Cytotect: 4%<br>No prophylaxis: 22.2%	Treatment well tolerated in all cases, without need of dose reduction

[a]The control group in this study reported rates of CMV disease but not CMV infection and was therefore not included in the meta-analysis [42].

[b]Article notes treatment for either 6 or 10 weeks following transplantation [53].

AE, adverse event; ALG, anti-lymphocyte globulin; ATG, anti-thymocyte globulin; AZA, azathioprine; CMV, cytomegalovirus; CMVIG, cytomegalovirus-directed hyperimmunoglobulin; CsA, cyclosporine A; CY, cyclophosphamide; D−, CMV-seronegative donor; D+, CMV-seropositive donor; ELISA, enzyme-linked immunosorbent assay; GVHD, graft-versus-host disease; HSCT, hematopoietic stem cell transplantation; IgG, immunoglobulin G; IgM, immunoglobulin M; IV, intravenous; MMF, mycophenolate mofetil; MTX, methotrexate; NR, not reported; OKT3, muromonab-CD3; PCR, polymerase chain reaction; R−, CMV-seronegative recipient; R+, CMV-seropositive recipient; TBI, total body irradiation.

Table S2. CMV infection rates in studies including high-risk (D+/R−) SOT patients only.

	CMVIG group		Control group	
Study	n/N	CMV infection rate, % (95% CI)	n/N	CMV infection rate, % (95% CI)
Varga et al., 2005 [54]	38/83	45.8 (34.8–57.1)	31/64	48.4 (35.8–61.3)
Pakkala et al., 1992 [42]	21/30	70.0 (50.6–85.3)	N/A[a]	N/A[a]
Snydman et al., 1987 [50]	6/24	25.0 (9.8–46.7)	15/35	42.9 (26.3–60.6)
Tenschert et al., 1993 [53]	4/18	22.2 (6.4–47.6)	18/18	100 (81.5–100)
Werner et al., 1993 [55]	38/174	21.8 (15.9–28.7)	N/A	N/A
Snydman et al., 1991 [51]	8/36	22.2 (10.1–39.2)	N/A	N/A
Boland et al., 1993 [26]	7/14	50.0 (23.0–77.0)	7/14	50.0 (23.0–77.0)
Kathawalla et al., 1996 [35]	2/3	66.7 (9.4–99.2)	6/6	100 (54.1–100)
Snydman et al., 2001 [47]	17/39	43.6 (27.8–60.4)	27/47	57.4 (42.2–71.7)
Rabkin et al., 2001 [43]	6/7	85.7 (42.1–99.6)	8/15	53.3 (26.6–78.7)

[a]The control group in this study reported rates of CMV disease but not CMV infection and was therefore not included in the analysis.

CI, confidence interval; CMV, cytomegalovirus; CMVIG, CMV-specific hyperimmunoglobulin; D+, CMV-seropositive donor; N/A, not applicable; R−, CMV-seronegative recipient.

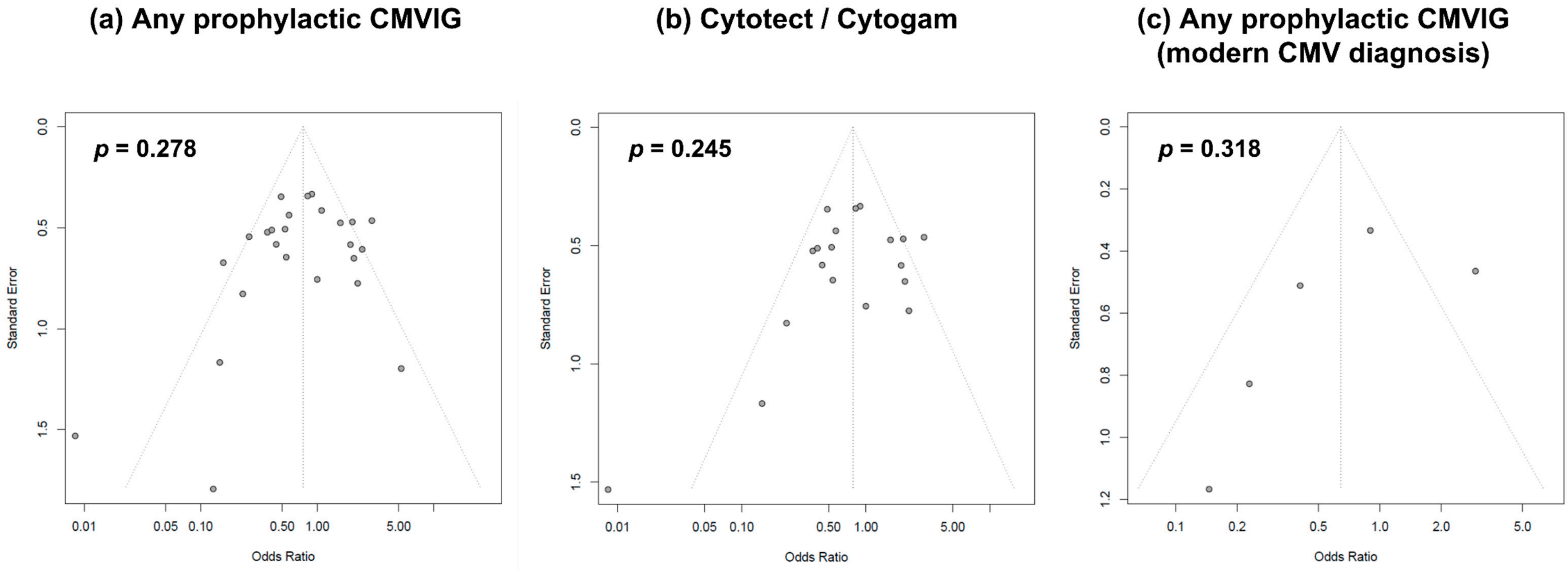

Figure S1. Funnel plots of the meta-analysis of studies considering any prophylactic CMVIG (**a**), Cytotect/Cytogam (**b**) and any prophylactic CMVIG in the era of modern CMV diagnosis, i.e., based on pp65 antigenemia and/or CMV DNAemia (**c**). Potential publication bias was assessed using funnel plots, considering studies with CMVIG and control arms. Funnel plots' asymmetry was evaluated using the Egger's test. The Egger's test *p*-values are indicated within each plot. No publication bias was evidenced in all conducted analyses ($p > 0.05$).
